# Explosive Mixtures

**Published:** 1885-03

**Authors:** 


### EXPLOSIVE MIXTURES.

Several instances are related in the *Deutsche Med. Zeit.*, of Sept., 1884, of injuries resulting from the explosion of compounds ordered in physicians' prescriptions. A gargle was ordered of chloride of potassium, chloride of iron, and glycerin. It was prepared, and five minutes later the bottle exploded in the purchaser's pocket, wounding him quite severely with the fragments of glass. A mixture of hypophosphite of lime, chlorate of potassium, and lactate of iron exploded, and nearly killed the prescription clerk who was compounding it. Even the simple trituration of calcium hypophosphite is dangerous; a young pharmacist was killed by an explosion which was caused by the shaking of a solution of this substance. Physicians not infrequently order a solution of chromic acid in glycerin. But when the acid is added quickly and all at once to the glycerin, a readily explosive substance like nitro-glycerin is formed. Chlorate of potassium, when mixed with tannin or muriate of morphia, often explodes. The combination of iodine and preparations of ammonia must be made cautiously, as iodide of nitrogen is formed, which explodes on the slightest touch. Indeed, one ought to be very careful in ordering and compounding mixtures in which easily reducible substances enter, such as the chlorates, the hypophosphites the nitrates, preparations of iodine or ammonia, chromic acid, glycerin, permanganate of potash, etc. If physicians would but remember the danger of explosions in preparing such compounds, they would less often put the lives of druggists and of their patients in jeopardy.

